# Verification of cardiac mechanics software: benchmark problems and solutions for testing active and passive material behaviour

**DOI:** 10.1098/rspa.2015.0641

**Published:** 2015-12-08

**Authors:** Sander Land, Viatcheslav Gurev, Sander Arens, Christoph M. Augustin, Lukas Baron, Robert Blake, Chris Bradley, Sebastian Castro, Andrew Crozier, Marco Favino, Thomas E. Fastl, Thomas Fritz, Hao Gao, Alessio Gizzi, Boyce E. Griffith, Daniel E. Hurtado, Rolf Krause, Xiaoyu Luo, Martyn P. Nash, Simone Pezzuto, Gernot Plank, Simone Rossi, Daniel Ruprecht, Gunnar Seemann, Nicolas P. Smith, Joakim Sundnes, J. Jeremy Rice, Natalia Trayanova, Dafang Wang, Zhinuo Jenny Wang, Steven A. Niederer

**Affiliations:** 1 Department of Biomedical Engineering, King's College London, London, UK; 2 Thomas J. Watson Research Center, IBM Research, Yorktown Heights, NY 10598, USA; 3 Department of Physics and Astronomy, Ghent University, Ghent, Belgium; 4 Institute of Biophysics, Medical University of Graz, Graz, Austria; 5 Institute of Biomedical Engineering, Karlsruhe Institute of Technology, Karlsruhe, Germany; 6 Department of Biomedical Engineering and Institute for Computational Medicine, Johns Hopkins University, Baltimore, MD 21218, USA; 7 Auckland Bioengineering Institute, University of Auckland, Auckland, New Zealand; 8 Department of Structural and Geotechnical Engineering, Pontifica Universidad Católica de Chile, Chile; 9 Center for Computational Medicine in Cardiology, Institute of Computational Science, Università della Svizzera italiana, Lugano, Switzerland; 10 School of Mathematics and Statistics, University of Glasgow, Glasgow, UK; 11 Department of Engineering, Nonlinear Physics and Mathematical Modeling Lab, University Campus Bio-Medico of Rome, Rome, Italy; 12 Interdisciplinary Applied Mathematics Center, University of North Carolina at Chapel Hill, Chapel Hill, NC, USA; 13 Department of Engineering Science, University of Auckland, Auckland, New Zealand; 14 Simula Research Laboratory, Fornebu, Norway; 15 Civil and Environmental Engineering Department, Duke University, Durham, NC 27708-0287, USA

**Keywords:** cardiac mechanics, verification, benchmark, VVUQ

## Abstract

Models of cardiac mechanics are increasingly used to investigate cardiac physiology. These models are characterized by a high level of complexity, including the particular anisotropic material properties of biological tissue and the actively contracting material. A large number of independent simulation codes have been developed, but a consistent way of verifying the accuracy and replicability of simulations is lacking. To aid in the verification of current and future cardiac mechanics solvers, this study provides three benchmark problems for cardiac mechanics. These benchmark problems test the ability to accurately simulate pressure-type forces that depend on the deformed objects geometry, anisotropic and spatially varying material properties similar to those seen in the left ventricle and active contractile forces. The benchmark was solved by 11 different groups to generate consensus solutions, with typical differences in higher-resolution solutions at approximately 0.5%, and consistent results between linear, quadratic and cubic finite elements as well as different approaches to simulating incompressible materials. Online tools and solutions are made available to allow these tests to be effectively used in verification of future cardiac mechanics software.

## 1. Introduction

Computational models of the heart are increasingly used to improve our understanding of cardiac physiology, including the effect of specific genetic changes and animal models of disease [1–3]. In addition, patient-specific models are being developed to predict and quantify the response of clinical interventions, identify potential treatments and evaluate novel devices [4,5]. For this purpose, a large number of independent simulation codes have been developed, ranging from open-source software and commercial products to a range of closed-source codes specific to individual research groups [6–8]. The shift of cardiac models from a research tool to a potential clinical product for informing patient care will bring cardiac models into the remit of clinical regulators. With this transition comes the requirement for improved coding standards. The move towards higher standards of accuracy and reproducibility is mirrored in many other fields of scientific computation [9]. A recent report by the National Research Council on the verification, validation and uncertainty quantification of scientific software addressed some of these issues with the aim of improving processes in computational science [10]. They define three specific categories of interest:

— erification. Determining how accurate a computer program solves the equations of a mathematical model.
— alidation. Determining how well a mathematical model represents the real world phenomena it is intended to predict.
— ncertainty quantification. The process of quantifying uncertainties associated with calculating the result of a model.

In cardiac modelling, uncertainty quantification has long been part of the accepted set of techniques, both in parameter sensitivity studies and studying the effects of biological variability. Approaches to uncertainty quantification include sensitivity analysis, visualization of parameter sweeps and the use of regression techniques [11–14]. More formal approaches have also been applied, including quantification of variability in high-throughput experiments and propagation of this variability in models, and high-order stochastic collocation methods to analyse variability in high-throughput ion channel data [15,16].

The variability and uncertainty of biological systems create significant challenges for validating computational models [9,17]. Historical data from the experimental literature cover a wide range of conditions with respect to temperature, species, genetic strain and other methodological detail [18]. As comprehensive and consistent experimental datasets are rarely available, the data most useful for validating predictions are nearly always used for constraining parameters and developing the model.

Verification is the process of determining a code's accuracy in solving the mathematical model it implements. This is an area that has also been widely recognized in high-stakes fields such as aeronautics, nuclear physics and weather prediction [17,19,20]. Until recently, verification has had a limited role in cardiac modelling. More recently, concerted verification efforts have been made in the area of cardiac electrophysiology solvers. These include an N-version benchmark now being used routinely [21], analytic solutions becoming available [22] and more benchmark tests currently being organized to expand these tests to cover more complex electrophysiological phenomena.

Similar domain-specific verification tests have been lacking for simulating cardiac mechanics. The heart has unique mechanical properties, including a contractile force generated by the tissue itself, and complex nonlinear and anisotropic material features. There are a range of analytic solutions which are commonly used in testing the correctness and convergence of solid mechanics software, most notably Rivlin's problems on torsion, inflation and extension of an incompressible isotropic cylinder [23]. Although these analytic solutions for solid mechanics problems help in verifying the correctness of mechanics codes, these typically do not test several crucial aspects specific to the simulation of cardiac mechanics. First, these problems with analytic solutions tend to be limited to isotropic material properties, whereas cardiac material is typically modelled as transversely isotropic or orthotropic. Second, complex pressure boundary conditions, in which both the area and orientation of the surface changes, are poorly tested. Third, active contraction of tissue is not tested, while it is the driving force in a simulation of cardiac function. As a result, simulation codes in the field are often under-verified, and a standard problem set is lacking. Similar limitations to using simple test problems with available analytic solutions were encountered in the field of computational fluid dynamics, which has a long history of verification and validation [19]. Lessons from this field include extending verification efforts to include benchmarks of carefully defined complex problems, and direct comparisons with experiments tailor-made for simulation validation. A complementary strategy for investigating reproducibility in the field of cardiac mechanics was the recent STACOM challenge [24], which asked participants to predict left-ventricular deformation between two given magnetic resonance imaging (MRI) datasets. As this challenge left many aspects open to user choice, including mesh generation, boundary constraints and material models, and did not aim for a single consensus solution, it is less suitable as a verification problem.

This report presents a set of three problems for the validation of cardiac mechanics software, along with an N-version benchmark of 11 different implementations. We have defined a series of benchmark problems that can be solved by typical cardiac mechanical simulators, with features that are important for solving cardiac mechanics problems.

## 2. Methods

We propose to verify cardiac mechanics codes using an N-version benchmark. For this approach to be effective, we need to ensure a large enough number of participants to achieve a community consensus for the solution, while ensuring that the test problems cover the salient properties of the codes. The cardiac mechanics benchmark problems should be simple enough to be clearly and unambiguously communicated, whereas complex enough to test important aspects of software codes not routinely tested using other methods. To ensure that any differences in solutions are due to differences in the implementation and not owing to ambiguities in the model definition or the use of different image processing tools, we use analytic descriptions for the geometry in all problems. However, we have not required the use of a specific numerical method, finite-element basis type or approach to modelling incompressibility, to maximize the number of potential participants. We have created a set of three different problems, each testing different aspects important to solving cardiac mechanics. The first problem uses a simple beam geometry with a typical cardiac constitutive law, testing the correct implementation of the governing equations, material properties and pressure boundary conditions changing with the deformed surface orientation and area. The second problem is independent of fibre direction and uses isotropic material properties, but tests a more complex left ventricular geometry. Finally, the third problem uses an identical geometry to the second problem, but adds a varying fibre distribution and active tension.

The free choice of numerical method and basis types poses challenges for comparing results and solution formats. As a compromise, the VTK file format is used for data output and processing, as this format is already in common use, several participants had built-in support for it in their software, and there is an extensive application program interface (API) for reading and processing results [25].

### (a) Solid mechanics theory and notation

We start with a brief overview to the theory of solid mechanics to introduce notation and concepts referred to in the problem description. We denote the undeformed location in Cartesian coordinates of a point as **X** and the deformed position as **x**=**x**(**X**). The deformation gradient is defined as **F**=∂**x**/∂**X**, and $\text{E}=\frac{1}{2}({\text{F}}^{T}\text{F}-\text{I})$ is the Green–Lagrange strain tensor. The governing equations for the deformation of an incompressible solid in steady-state equilibrium can be stated as

2.1 div σ=0(balance of momentum)

and

2.2 under the constraint J=1(incompressibility),

where J=det(F) and ***σ*** is the Cauchy stress tensor which is derived from a strain energy function *W*(**E**) by

2.3 $$J{\text{F}}^{-1}\sigma {\text{F}}^{-T}=\text{T}=\frac{\partial W}{\partial \text{E}},$$

where **T** is the second Piola–Kirchhoff stress tensor. Apart from these basic governing equations, theory is dependent on the numerical approach. Further derivation usually proceeds by the principle of virtual work to derive a finite-element weak form. Reviews of modelling mechanics and finite-element approaches can be found in the literature, e.g. Holzapfel [26] or Bonet & Wood [27]. Regardless of the discretization used, the equations are both inherently nonlinear, and additional nonlinearity is introduced when using a nonlinear strain energy function *W*(**E**), which is the norm in cardiac mechanics simulations. To maximize the number of participants and encourage a wide range of solutions, we have made no particular requirement or recommendation for specific numerical approaches in defining the benchmark problems.

### (b) Constitutive law

Cardiac tissue consists of a mesh of collagen with cardiac muscle cells, or ‘cardiomyocytes’. Cardiomyocytes are approximately 100×10×10 μ*m* in size, with a distinct long axis, often referred to as the ‘fibre direction’. Taking into account, the fibre direction leads to models with a transversely isotropic constitutive law [28–30]. In addition, laminar sheets have been identified, with more collagen links between cells in a sheet, compared with between sheets. Taking these sheets into account gives rise to an orthotropic material law [31–34]. However, histological examination shows that while sheets are clearly present in the mid-myocardium, their presence is not uniform throughout the myocardial wall [35]. In addition, defining a problem with orthotropic material properties requires a more complex problem description, and not all participants have software that supports simulating this kind of material.

For the benchmark problems, we use the transversely isotropic constitutive law by Guccione *et al.* [28]. It was anticipated that this constitutive law would be the most widely implemented by potential participants because it is relatively simple and has been widely used in cardiac modelling. Its strain energy function is given by

2.4 $$W=\frac{C}{2}(e^{Q}-1)$$

and

2.5 $$Q=b_{f}E_{11}^{2}+b_{t}\left(E_{22}^{2}+E_{33}^{2}+E_{23}^{2}+E_{32}^{2}\right)+b_{fs}\left(E_{12}^{2}+E_{21}^{2}+E_{13}^{2}+E_{31}^{2}\right),$$

where *E*_*ij*_ are components of the Green–Lagrange strain tensor **E** in a local orthonormal coordinate system with fibres in the **e**_1_-direction, and where *C*,*b*_*f*_,*b*_*t*_,*b*_*fs*_ are the material parameters which will be defined for each of the three problem separately. In all problems, the material is fully incompressible, i.e. *J*=1 as stated in equation (2.2). Please note that in all problems the direction of the pressure boundary condition changes with the deformed surface orientation, and its magnitude scales with the deformed area. There were no restrictions on the methods used to satisfy incompressibility, and participants used both Lagrange multiplier methods as well as quasi-incompressibility approaches with penalty functions to satisfy this constraint.

### (c) Problem descriptions

The following sections give a complete and reproducible description of each of the three benchmark problems as distributed to the participants. In addition to an incompressible large deformation elasticity formulation and a description of the constitutive law, five additional components were required for a reproducible problem definition: a reproducible problem definition requires five additional parts: a problem geometry, the material parameters (*C*,*b*_*f*_,*b*_*t*_,*b*_*fs*_), a full description of the fibre direction throughout the geometry, the Dirichlet boundary conditions and the applied pressure boundary conditions. The three problems each test different aspects important to cardiac mechanics solvers. The first problem is the simulation of a deforming rectangular beam. This problem tests pressure-type forces whose directions change with the deformed surface orientation, and the correct implementation of fibre directions changing with the deformation, the transversely isotropic constitutive law, and Dirichlet boundary conditions. This problem uses a simple mesh geometry, which makes it easier to quickly test new codes and provide an initial verification test. The second problem is the inflation of an ellipsoid with isotropic material properties. The problem tests the reproduction of a mesh geometry from a description, and a deformation pattern similar to cardiac inflation. The third problem is the inflation and active contraction of an ellipsoid with transversely isotropic material properties. The problem tests reproducibility of complex fibre patterns, and the implementation of active contraction, both important aspects of a cardiac mechanics solver. Using two problems on the same initial geometry allows benchmark participants to generate a mesh geometry and verify inflation first, before the source of potential errors is conflated with the implementation of active contraction and fibre directions. This is intended to make it easier to track down potential errors in an implementation.

#### (i) Problem 1: deformation of a beam

**Figure 1.**
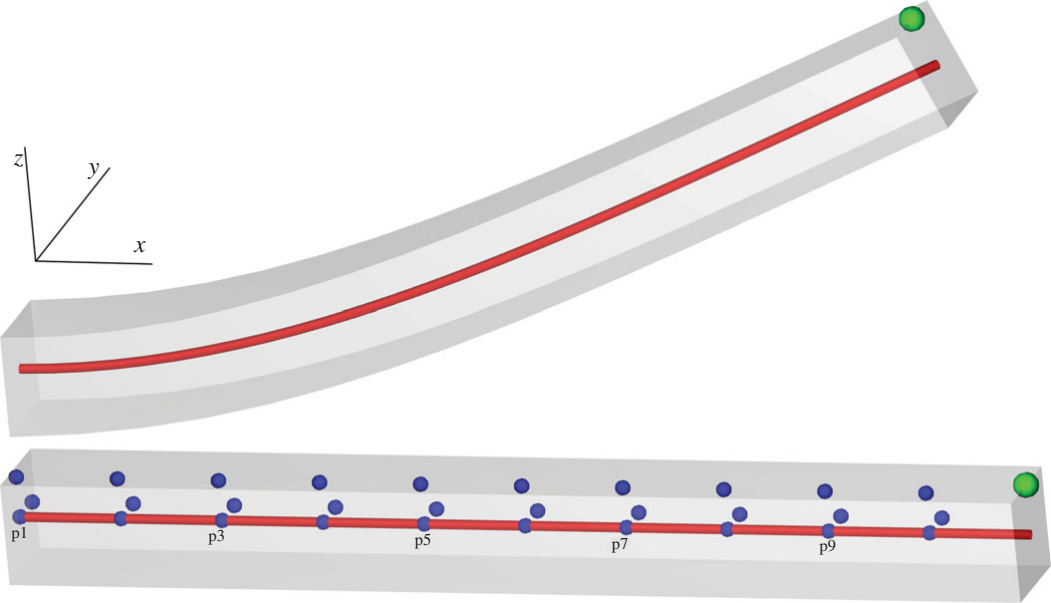
Problem 1.Deformation of a beam with the reference geometry (bottom) and an example solution (top). The green node indicates the position of results in figure 3, the red line indicates the line used for results in figure 4, and the blue points indicate the locations used in the strain calculations. (Online version in colour.)

Figure 1 shows the problem geometry and a representative solution.

*Geometry*: the undeformed geometry is the region *x*∈[0,10], *y*∈[0,1], *z*∈[0,1] mm.

*Constitutive parameters*: transversely isotropic, *C*=2 kPa,*b*_*f*_=8,*b*_*t*_=2,*b*_*fs*_=4.

*Fibre direction*: constant along the long axis, i.e. (1, 0, 0).

*Dirichlet boundary conditions*: the left face (*x*=0) is fixed in all directions.

*Pressure boundary conditions*: a pressure of 0.004 kPa is applied to the entire bottom face (*z*=0).

#### (ii) Problem 2: inflation of a ventricle

**Figure 2.**
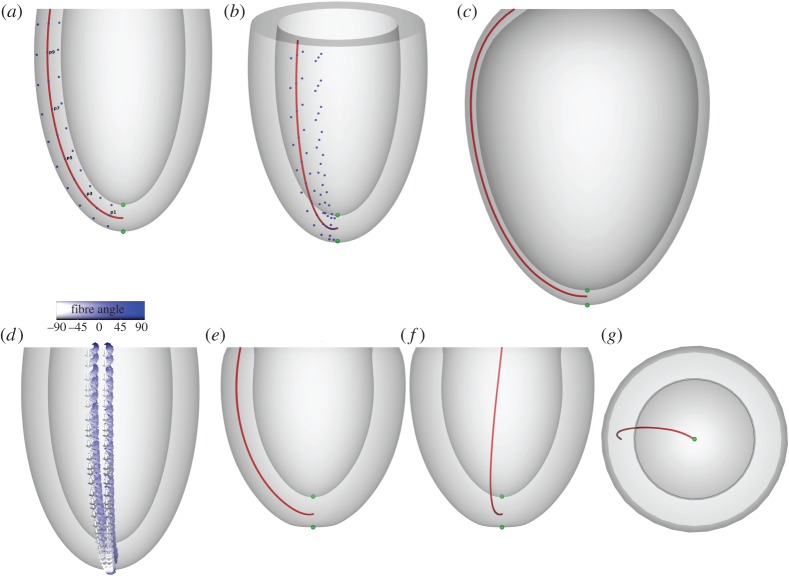
Problems 2 and 3. Panels (*a*,*b*) show the reference geometry for both problem 2 (inflation of a ventricle) and 3 (inflation and active contraction of a ventricle). The green nodes indicate the apical position used in results in figures 6 and 9, and the red line indicates the line used for results in figure 7 and 10. Blue nodes areused for strain calculations as described in §3, with panel (*a*) showing only nodes at *v*=0 and panel (*b*) showing both nodes at *v*=0 and *v*=*π*/10 used for circumferential strain calculations. Panel (*c*) shows an example solution to problem 2. Panel (*d*) shows the fibre directions used in problem 3, varying from −90° at the epicardium to +90° at the endocardium. Panels (*e*,*f*) show different side views of one example solution to problem 3, and panel (*g*) shows a view from the base. (Online version in colour.)

Figure 2 shows the problem geometry and an example solution.

*Geometry*: the undeformed geometry is defined using the parametrization for a truncated ellipsoid:

2.6 $$\text{x}=\left(\begin{array}{c} x\\ y\\ z \end{array}\right)=\left(\begin{array}{c} r_{s}\sin u\cos v\\ r_{s}\sin u\sin v\\ r_{l}\cos u \end{array}\right).$$

The undeformed geometry is defined by the volume between:

— the *endocardial surface* $r_{s}=7\;\mathrm{mm},r_{l}=17\;\mathrm{mm},u\in [-\pi ,-\arccos\frac{5}{17}],v\in [-\pi ,\pi ]$,
— the *epicardial surface* $r_{s}=10\;\mathrm{mm},r_{l}=20\;\mathrm{mm},u\in [-\pi ,-\arccos\frac{5}{20}],v\in [-\pi ,\pi ]$
— the *base plane* *z*=5 *mm* which is implicitly defined by the ranges for *u*.

*Constitutive parameters*: isotropic, *C*=10 kPa, *b*_*f*_=*b*_*t*_=*b*_*fs*_=1.

*Dirichlet boundary conditions*: the base plane (*z*=5 mm) is fixed in all directions.

*Pressure boundary conditions*: a pressure of 10 kPa is applied to the endocardial surface.

#### (iii) Problem 3: inflation and active contraction of a ventricle

*Geometry, Dirichlet boundary conditions*: identical to problem 2.

*Fibre definition*: fibre angles *α* used in this benchmark problem range from −90° at the epicardial surface to +90° at the endocardial surface. These angles were chosen to allow for easy visual inspection of generated fibre directions, despite being steeper than those measured in DTMRI experiments [36]. They are defined using the direction of the derivatives of the parametrization of the ellipsoid in equation (2.6)

2.7 $$\begin{array}{rl} f(u,v) & =n\left(\frac{d\text{x}}{du}\right)\sin\alpha +n\left(\frac{d\text{x}}{dv}\right)\cos\alpha ,\;\mathrm{where}\;n(\text{v})=\text{v}/∥\text{v}∥, \end{array}$$

2.8 $$\begin{array}{rl} \frac{d\text{x}}{du} & =\left(\begin{array}{c} r_{s}\cos u\cos v\\ r_{s}\cos u\sin v\\ -r_{l}\sin u \end{array}\right), \end{array}$$

2.9 $$\begin{array}{rl} \frac{d\text{x}}{dv} & =\left(\begin{array}{c} -r_{s}\sin u\sin v\\ r_{s}\sin u\cos v\\ 0 \end{array}\right), \end{array}$$

2.10 $$\begin{array}{rl} r_{s}(t) & =7+3t, \end{array}$$

2.11 $$\begin{array}{rl} r_{l}(t) & =17+3t \end{array}$$

2.12 $$\begin{array}{rl} \;\text{and}\;\;\alpha (t) & =90-180t, \end{array}$$

where *r*_*s*_, *r*_*l*_ and *α* are derived from the transmural distance *t*∈[0,1] which varies linearly from 0 on the endocardium and 1 on the epicardium. The apex (*u*=−*π*) has a fibre singularity which is common in cardiac mechanics problems. No specific approaches are prescribed for handling this singularity, and all approaches were considered acceptable.

*Constitutive parameters*: transversely isotropic, *C*=2 kPa, *b*_*f*_=8, *b*_*t*_=2, *b*_*fs*_=4.

*Active contraction*: the active stress is given by a constant, homogeneous, second Piola–Kirchhoff stress in the fibre direction of 60 kPa, i.e.

2.13 $$\text{T}={\text{T}}_{p}+T_{a}\text{f}{\text{f}}^{T},$$

where *T*_*a*_=60 kPa, **f** is the unit column vector in the fibre direction described above, and the passive stress **T**_*p*_=∂*W*/∂**E** as in equation 2.3.

*Pressure boundary conditions*: a constant pressure of 15 kPa is applied to the endocardium. As this is a quasi-static problem, participants are free to add active stress first, add pressure first or increment both simultaneously in finding a solution. Figure 2 shows the problem geometry and an example solution.

### (d) Participants

Table 1 lists the participants and the computational methods they used. Although there was no requirement to use a specific computational method, all participants used finite-element methods, as they are most common in the field of cardiac mechanics.

**Table 1.** Overview of methods and software used by participants of the mechanics benchmark. Superscripts in the ‘affiliations’ column refer to the contributing institution details as given on the title page. Details for open source code origins and availability are given below for groups who use publicly available code. The ‘method’ summarizes the type of finite-elements used, with ‘Q*x*’ referring to order *x* hexahedral elements, ‘P*x*’ to order *x* tetrahedral elements, and ‘Q*x*Q*y*’, ‘P*x*P*y*’ to order *x* elements for deformation and order *y* elements for the Lagrange multiplier. When two element types are listed, the first was used for problem 1, and the second for problems 2 and 3. I/D denotes the use of an approach with isochoric/deviatoric splitting of the deformation gradient.

code name	affiliation	type	references	method
Cardioid	IBM^2^	in-house	[8]	Q2Q1/P2P1, Lagrange multiplier, I/D
CardioMechanics	KIT^5^	in-house	[2]	P2, quasi-incompressible
CARP	Graz^1,4^	in-house	[37]	P1P0, quasi-incompressible, I/D
Elecmech	KCL^1^	in-house	[38,39]	Q3Q1, Lagrange multiplier
GlasgowHeart-IBFE	Glasgow^10,12^	in-house	[40]	Q1, IB/FE^a^
Hopkins-MESCAL	Hopkins^6^	in-house		Q1P0, Lagrange multiplier
LifeV	Duke^15^	open source^b^	[41,42]	P2, quasi-incompressible
MOOSE-EWE	USI^9^	mixed^c^	[43]	Q2Q1/P2P1, Lagrange multiplier, I/D
OpenCMISS	Auckland^3,7,13^	open source^d^	[6]	Q3Q1 (hermite), Lagrange multiplier, I/D
Simula-FEniCS	Simula^14^	open source^e^	[44,45]	P2P1^f^, Lagrange multiplier, I/D
PUC-FEAP^g^	PUC^8,11^	open source	[46]	Q1P0, Lagrange multiplier, I/D

^a^IB/FE indicates the immersed boundary method using a finite-element mechanics model, and used the open-source IBAMR software available at https://github.com/IBAMR/IBAMR.

^b^LifeV was developed by EPFL and available at http://github.com/lifev.

^c^MOOSE is open source and available at http://www.mooseframework.org/, EWE is an in-house application using MOOSE.

^d^OpenCMISS available at http://www.opencmiss.org.

^e^FEniCS was developed by Simula and is available at http://fenicsproject.org, with problem-specific source code available at https://bitbucket.org/peppu/mechbench.

^f^FEniCS using two-dimensional elements in problems 2 and 3.

^g^PUC-FEAP: no solution submitted for problems 2 and 3, FEAP available at http://www.ce.berkeley.edu/projects/feap/.

## 3. Results

Here, we analyse and compare the submitted solutions with the benchmark problems. In terms of three-dimensional deformation as visualized, the submitted solutions are typically indistinguishable, so such visualizations are not included for all solutions. There are no analytic solutions to the problems, which limits the use of typical convergence analysis. In addition, the range of different finite-element basis types used result in further challenges in processing data and comparing solutions.

To analyse and compare results, we use the API provided by VTK [25].^1^ Participants were requested to provide meshes for the deformed and undeformed configurations in the VTK file format. Where a basis type was not supported by VTK, specifically on cases using cubic-order elements, solutions were interpolated to a compatible VTK element type. Our strategy for comparing solutions is based on a method for determining the deformed location of specific points in the submitted solutions for all participants. Using the VTK API, we locate the element containing a specific point in the undeformed mesh provided by a participant, along with the local parametric coordinates within that element. We use these local coordinates to locate the corresponding deformed point in the same element of the deformed geometry provided. This process allows us to track displacements in a wide variety of element types.

To calculate strain *S*_*i*_, we track changes in the distance between pairs of *n* points with coordinates $X_{1}^{i}$ and $X_{2}^{i}$ in the undeformed finite-element geometries and coordinates $x_{1}^{i}$ and $x_{2}^{i}$ of the deformed geometry, where i=0,1,…,n. We use a finite difference scheme to determine the strain

3.1 $$S_{i}=\left(\frac{∥x_{1}^{i}-x_{2}^{i}∥}{∥X_{1}^{i}-X_{2}^{i}∥}-1\right)\times 100\%.$$

For the beam problem, we use neighbouring points along the line (*x*,0.5,0.5) to calculate axial strain in the *x*-direction: $X_{1}^{i}=(i,0.5,0.5)$ and $X_{2}^{i}=(i+1,0.5,0.5)$, where *i*=0,1,…,8. For transverse strain, we use $X_{1}^{i}=(i,0.5,0.5)$, where *i*=0,1,…,9 and $X_{2}^{i}=(i,0.9,0.5)$ and $X_{2}^{i}=(i,0.5,0.9)$ for strain calculations in the *y*- and *z*-directions, respectively.

For the ellipsoidal problems, longitudinal, circumferential and radial strain are each calculated at the endocardium, epicardium and midwall. We use the parametrization in equations (2.6)–(2.12) and take the points along apex-to-base lines: *v*_*i*_=0, *u*_*i*_=*u*_1_+(*u*_2_−*u*_1_)/*n*_*u*_×(*i*+1)×0.95, where *u*_1_=−*π*, $u_{2}=-\arccos5/\left(17+3t\right)$, *n*_*u*_=10 and *i*=0,1,…,*n*_*u*_−1. These lines are taken along the endocardium (*t*=0.1), epicardium (*t*=0.9) and midwall (*t*=0.5). For longitudinal strain, we use pairs of neighbouring points along each line. For transmural strains, we use pairs of neighbouring endocardium-midwall, midwall-epicardium and endocardium–epicardium points to calculate radial strain at endocardium, epicardium and midwall. To calculate circumferential strain, the second point $X_{2}^{i}$ is derived by rotating the points at each myocardial layer by using *v*_*i*_=*π*/10 instead of *v*_*i*_=0. The points used for strain calculation are also shown in figures 1 and 2.

Overall, we perform three types of comparisons for each problem. First, we look at key points in the solution, which provides a crude but efficient measure of solution accuracy, and allows us to plot the accuracy of all solutions as a function of the number of degrees of freedom used to solve the problem. Second, we display the deformation of key lines through the mesh, which provides a more global measure of accuracy while still being easy to interpret and compare in a two-dimensional plot. Third, we calculate the strain measures described in this section to enable a more complex quantitative comparison of the deformation in each direction.

### (a) Problem 1

**Figure 3.**
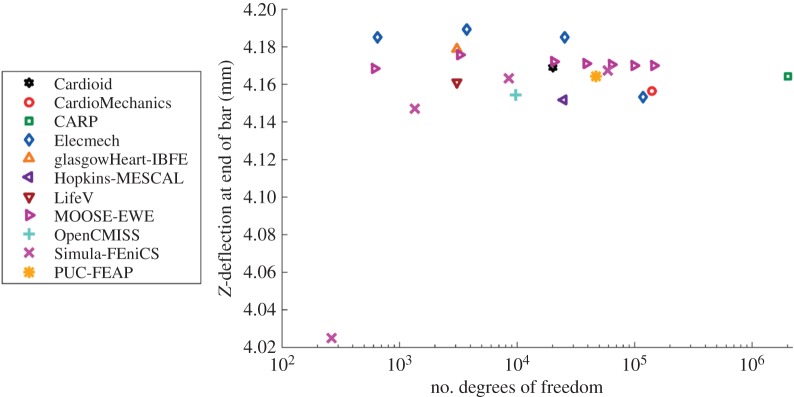
Problem 1: maximal deflection. Shown is the deformed location of the point (10,0.5,1). Results converge to a consensus solution as the number of degrees of freedom increases. Note that for the IB/FE method, only degrees of freedom in the solid mechanics problem are counted. (Online version in colour.)

**Figure 4.**
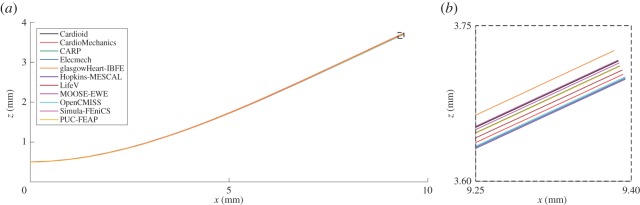
Problem 1: deformation of a line. Shown is the deformed location of the line (*x*,0.5,0.5) for each of the submitted solutions, with details for the end of the bar. (Online version in colour.)

**Figure 5.**
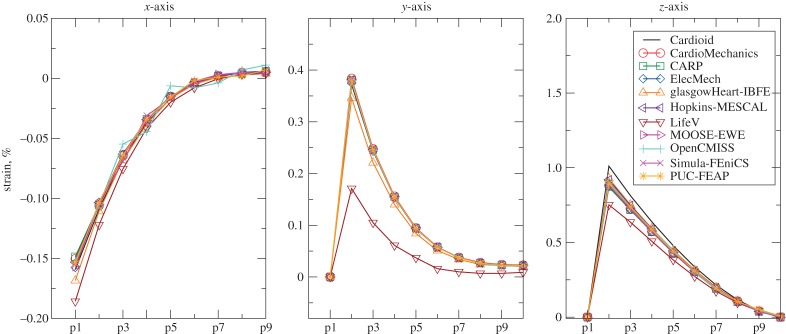
Problem 1: strain results. Plot of strain along the line in directions of *x*-, *y*- and *z*-axes. The index of points indicated on the horizontal axis increases as X=0,1,… and labels correspond to those given in figure 1. (Online version in colour.)

Figure 3 shows the maximal deflection of the beam across different solutions plotted against the number of degrees of freedom used, with the deformed position of a specific line at maximal deflection shown in figure 4. Figure 5 shows a comparison of strain measures in the submitted solutions. For both the strain measures and the deformed solution, only the solutions with most refined discretizations were used.

### (b) Problem 2

**Figure 6.**
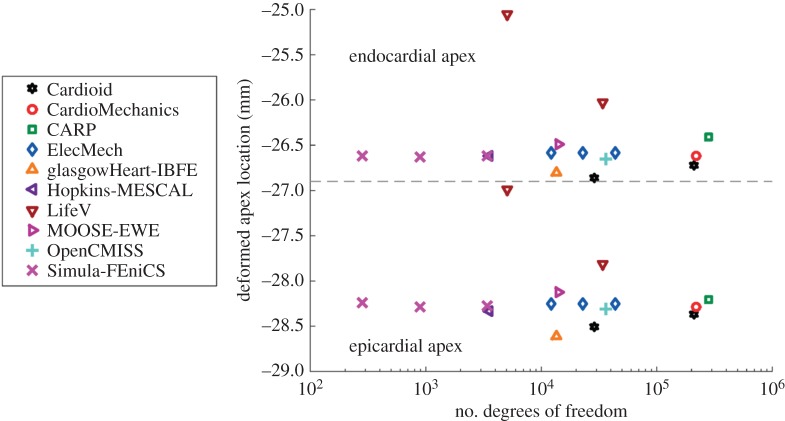
Problem 2: apex location. The dashed line separates results for the deformed positions of the apex at the endo- and epicardium, and the deformed position of the epicardium. (Online version in colour.)

**Figure 7.**
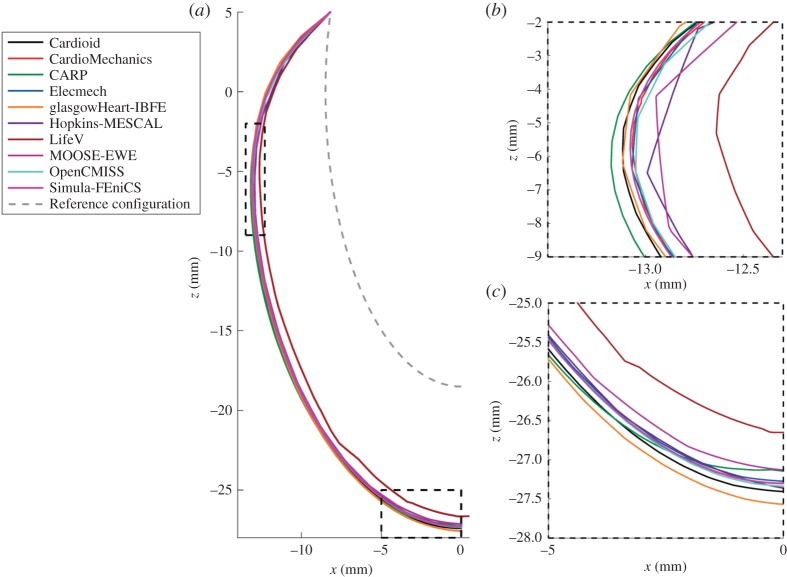
Problem 2: deformation of a line. Panel (*a*) shows the deformed location of a line in the middle of the ventricular wall (8.5sin⁡u,0,18.5cos⁡u), as shown in red in figure 2, with details of the apical region (*b*) and inflection point (*c*). (Online version in colour.)

**Figure 8.**
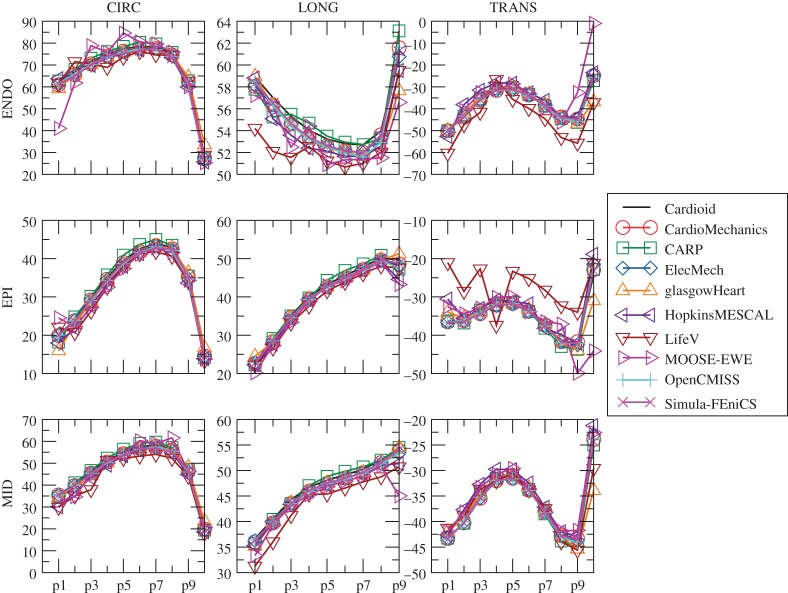
Problem 2: strain results. Plot of longitudinal (LONG), circumferential (CIRC) and radial (TRANS) strains at endocardium, epicardium and midwall. Index of points increases from the apex to the base, and labels correspond to those given in figure 2. (Online version in colour.)

Figure 6 shows the location of the endocardial and epicardial apex plotted against the number of degrees of freedom used. Figure 7 shows the deformed position of a line in the midwall from apex to base for all the submitted solutions, with details of the apex and inflection point. Figure 8 shows a comparison of strain measures for the submitted solutions.

### (c) Problem 3

**Figure 9.**
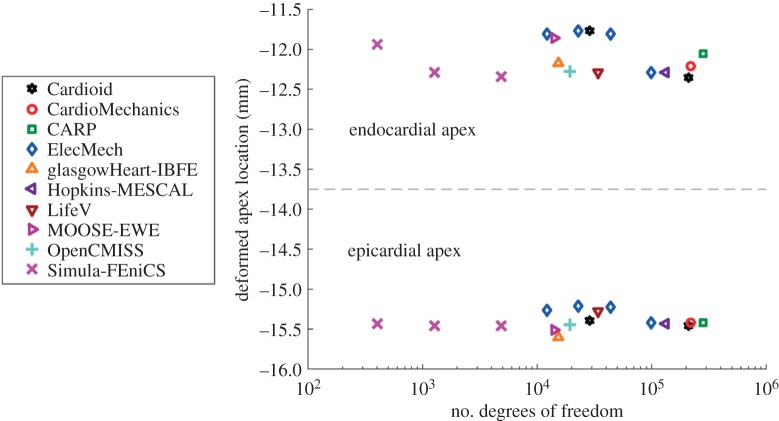
Problem 3: apex location. The dashed line separates results for the deformed positions of the apex at the endo- and epicardium. (Online version in colour.)

**Figure 10.**
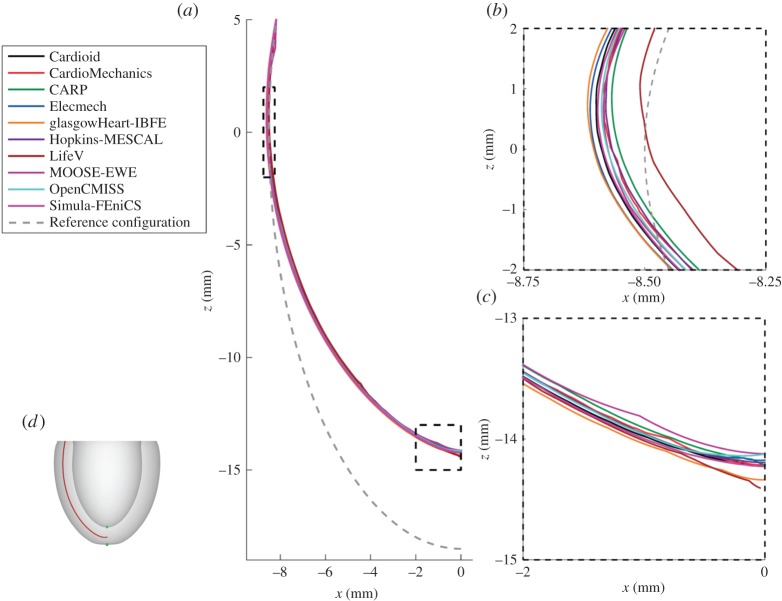
Problem 3: deformation of a line. Panel (*a*) shows the deformed location of the line in the middle of the ventricular wall (8.5sin⁡u,0,18.5cos⁡u) (shown in red in figure 2*e*, replicated here in panel *d*), with details of the apical region (*b*) and inflection point around *z*=0 (*c*). (Online version in colour.)

**Figure 11.**
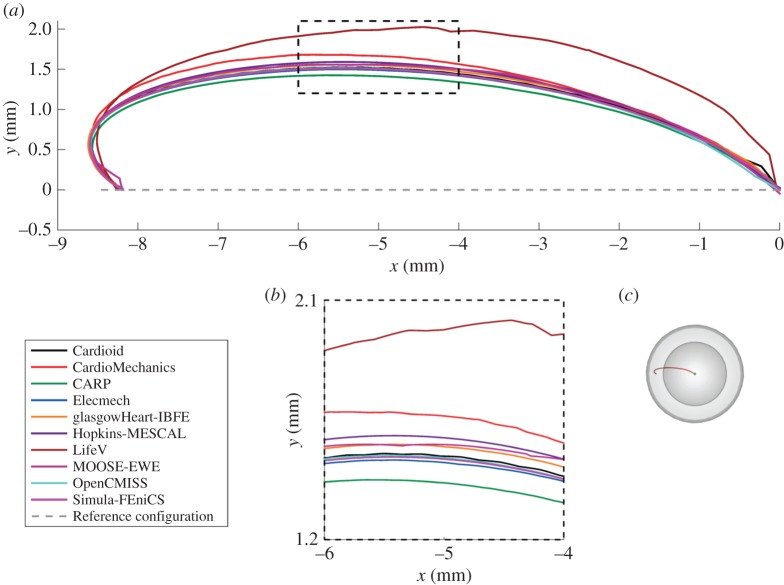
Problem 3: deformation of a line show twist. Shown is the deformed location of the line *t*=0.5 (*a*) (in the perspective shown figure 2*g*, replicated here in panel *c*), with details of the region around *x*=5 (*b*). Note that the line for the reference configuration starts at *x*≈−8.18 like the deformed line, but overlaps itself on the segment *x*∈[−8.18,−8.5] owing to the perspective shown. (Online version in colour.)

**Figure 12.**
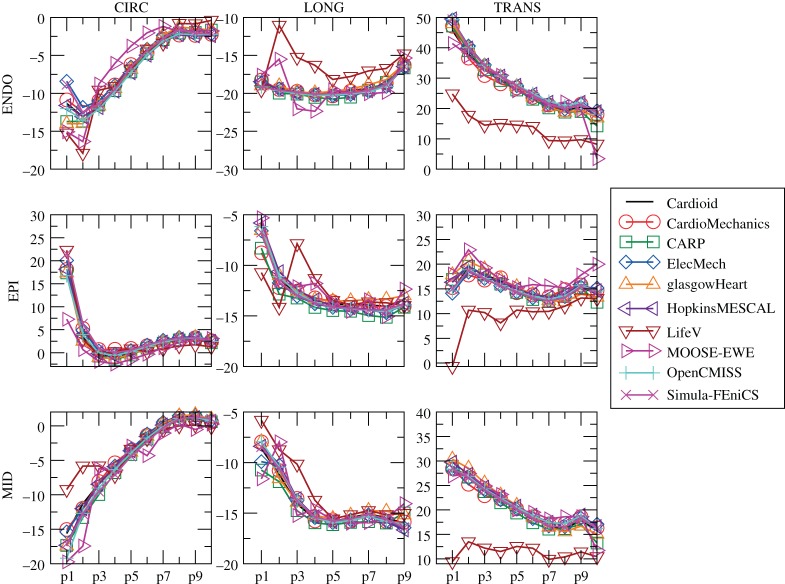
Problem 3: strain results. Plot of longitudinal (LONG), circumferential (CIRC) and radial (TRANS) strains at endocardium, epicardium and midwall. Index of points increases from the apex to the base, and labels correspond to those given in figure 2. (Online version in colour.)

Figure 9 shows the location of the endocardial and epicardial apex plotted against the number of degrees of freedom used. Figure 10 shows the deformed position of a line in the midwall from apex to base for all the submitted solutions, and figure 11 shows the position of this same line as viewed from the top, comparing results for the twisting motion of the ventricle under active contraction. Details are provided of several key regions to highlight small differences between solutions. Figure 12 shows a comparison of strain measures for the submitted solutions.

## 4. Discussion

This study presented a set of benchmark problems and an in-depth evaluation of 11 different cardiac mechanics codes, each submitting between one and four solutions for the three benchmark problems. The results, processing tools and MATLAB scripts for mesh generation, are made available online to assist in the verification of additional software in the field.^2^

In addition to verifying a basic solid mechanics solver, the benchmark problems test several aspects of software specific to cardiac mechanics. First, all three problems test pressure boundary conditions that depend on the deformed surface orientation and area. This has typically been the only type of external force applied to the heart in physiological cardiac simulations, although some more recent work implements contact mechanics with the pericardium [2], or spring boundary conditions to simulate contact with soft material near the apex. Second, the problems test a commonly used transversely isotropic constitutive law, as well as a complex fibre distribution. Fibre directions are most commonly stated in terms of ‘fibre angles’, and routinely visualized in the literature. However, they are rarely described accurately enough to ensure reproducibility, as the conversion from fibre angles to fibre vectors can rely on details of mesh geometry and implementation of local coordinate systems and orthogonalization. Specifically, fibre orientations are often defined with respect to local finite-element mesh coordinates, but mesh personalization tools vary, and there is no guarantee that these local coordinates unambiguously align with the apical-basal or circumferential directions. As a result, reproducible fibre directions are rarely given in studies on cardiac mechanics. In this study, the use of an exact mathematical description of fibre directions provided an unambiguous anatomical description and demonstrates that this description is sufficient for different groups to reproduce a solution. Third, we have tested the inclusion of contractile forces. Although these contractile forces are arguably the most important factor driving cardiac deformation, there have been no tests of its correct implementation proposed so far. The third benchmark problem tests this aspect and reproduces the typical twisting motion with apical–basal shortening of the ventricle in systole. Overall, the current problems aim to strike a balance between problem complexity and testing aspects that are important in cardiac mechanics, but currently not routinely tested. At the same time, they were designed to be simple enough to run relatively quickly, increasing their utility in rapid verification of software.

In this report, we have used a variety of methods to compare the submitted solutions. Showing deformation results for a few key points plotted against the number of degrees of freedom is a concise way of comparing a large number of solutions. They also provide a quick verification test for new software codes, as comparison of a single point for each problem can quickly reveal incorrect solutions. A drawback of this technique is that the selected points of comparison may not be representative of the overall accuracy of a solution. Especially in problem 3, errors localized at the apex show up disproportionately, and are not always representative of the accuracy elsewhere. Therefore, we have highlighted both regions close to the apex as well as closer to the base. Plotting the deformation of lines through the simulation domain as in figures 4, 7 and 10 shows more information, but can quickly result in solutions overlapping in visualizations. Finally, we have plotted strain in different directions, and at different locations, for the submitted solutions. For problem 1, despite the large deformation of the beam, relatively small strains on the order of 1% were observed (figure 5). The largest strains and largest discrepancies between solutions are located near the Dirichlet boundary conditions at *x*=0. Thus, this strain test is useful to reveal obvious errors in implementation or to reveal shortcomings in discretization such as volumetric locking by linear finite elements. A potential explanation for differences in strain between submitted solutions is the low number of degrees of freedom used by some groups. Strain results for problem 2 (figure 8) are most consistent across submitted solutions, suggesting that this was the least challenging test. Problem 3 was the most challenging test, and a number of participants submitted several revised solutions before the close agreement in strains shown in figure 12 was achieved. These visualizations are richer, comparing the solutions across a wider area, and more clearly show local similarities and differences. However, they require a larger number of plots to show, and are more difficult to interpret and define in a reproducible way. Requiring only solutions of the deformation increased participation, given the varied capabilities of the software used by different participants, but limited the range of possible comparison methods. Further comparisons would have been possible by requiring participants to provide Cauchy–Green strain, or deformed fibre directions in problem 3. Although it is theoretically possible to obtain these metrics using finite-difference methods, in practice, we found this approach not robust enough in the VTK implementation, leading us to use more global strain metrics.

In total, 11 different groups submitted solutions to the benchmark problems. Although the choice of computational methods was left open, all participants used finite-element methods to solve the problems. Most commonly used were quadratic-order tetrahedral elements and linear hexahedral elements. In addition, to these standard solution methods, several unique approaches were applied in solving the benchmark problems. First, problem 2 and 3 were rotationally symmetric, and participants from Simula Research Laboratory exploited this feature by solving these problems using two-dimensional elements, allowing very high-resolution solutions. Second, participants from the University of Glasgow applied the immersed boundary method with finite-element extension (IB/FE) developed for their coupled fluid-structure implementation [40]. The IB/FE method is designed for dynamic fluid-structure analyses rather than for the quasi-static analyses considered in this study, but its inclusion in the study highlights the usefulness of the benchmark problems in verification of both static and dynamic solvers. Overall, there was broad agreement between participants, with typical differences in deformation at approximately 1% (figures 3, 7, 6, 9, 10). The largest differences that were encountered were attributed to

— under-converged results, e.g. the high discrepancy between solutions with a few hundred degrees of freedom and those with over 10^5^ in problem 1 (figure 3). However, in problems 2 and 3, the solutions with larger difference from consensus solutions were not necessarily those with the fewest degrees of freedom used. Specifically, the use of two-dimensional elements exploiting problem symmetry by Simula Research Laboratory achieved excellent accuracy with very low degrees of freedom used. Nevertheless, the largest error for those using three-dimensional elements appears for LifeV, who use the fewest degrees of freedom.
— the use of a passive isotropic region near the apex in problem 3. This clearly shows differences in apical strain (figure 10), especially for earlier submitted results using a relatively large region with passive material properties, but is also still visible for several results in the final set. However, despite differences at the apex, results were consistent with other codes in the basal regions.

In addition, several participants reported potential stability problems. Fung-type constitutive laws, including the one used in this benchmark, can become unstable depending on the material parameters and loading conditions [47]. This was reported to lead to potential stability issues in problem 1, although all participants managed to solve to the load specified in the problem description. Participants from Simula Research Laboratory noted that problem 2 can fail to solve at around 3 kPa pressure when using P2P1 elements unless volumetric–deviatoric splitting of the deformation gradient is used. Similar stability problems were reported by the PUC group at around 10.5 kPa using Q1P0 elements, despite the use of volumetric–deviatoric splitting. The benchmark also tests the ability of software to handle problems with different properties in terms of their stiffness matrix, which potentially affects solver convergence. Specifically, problems 2 and 3 have a symmetric stiffness matrix owing to the boundary of the region where pressure is applied being completely fixed, whereas problem 1 has an asymmetric stiffness matrix (cf. Bonet & Wood [27], §6.5.2).

As the first significant benchmark in the field of cardiac mechanics, we have aimed to strike a balance between maximizing participation and testing more complex aspects of cardiac mechanical simulations. As such, this initial study has a number of potential limitations. First, problem 3 adds both fibre directions and active contraction, whereas an additional problem could test only passive properties, leading to more fine-grained verification. However, in the context of limiting the number of problems to be solved, we found it more important to introduce mesh geometry and fibre directions in separate problems, as both are difficult to unambiguously describe and reproduce. For problems 2 and 3, the curved geometry combined with the free choice of elements and meshing strategy, means that not all points in the problem domain appear in each mesh. This limits comparison with regions present in all submitted solutions, and specifically prevents comparison of solutions near the edge of the problem domain. In addition, the limited support for higher-order elements in VTK required interpolating cubic-order solutions to linear elements, which has the potential for introducing additional error. Finally, although this benchmark tests a number of important aspects specific to cardiac mechanics, future benchmarks could test several more detailed aspects not touched on by these benchmark problems. Specifically, an important aspect that was not tested is the use of time-dependent solutions of the cardiac cycle with heterogeneous activation patterns. These whole cycle simulations also require specialized numerics required to deal with length- and velocity-dependence of cardiac tension, and techniques for coupling to hemodynamic models, which were not tested in the current benchmark. Other aspects could involve using non-symmetric geometries, biventricular models or including the personalization of a mesh from a segmented image. In the context of the increase in patient-specific modelling, a verification of with local heterogeneities in material properties and contractile force, as observed in ischaemia, would be particularly important.

In conclusion, the development of a set of benchmark problems for simulating cardiac mechanics is an important step in the process of verification of cardiac modelling software. These results now provide us a standard and reproducible set of problems to drive forward the development and verification of simulation platforms and numerical methods tailored to the domain-specific characteristics of cardiac mechanics modelling.

## Footnotes

1 Available at http://www.vtk.org/download/.

2 Repository of results: http://www.bitbucket.org/sander314/mechbench.

## Data accessibility

All submitted solutions are available on the repository http://www.bitbucket.org/sander314/mechbench.
